# Choral Singing and its Effect on Wellness Among Older Adults: Engagement, Self-Expression, and Connection

**DOI:** 10.1093/geroni/igaf122.2473

**Published:** 2025-12-31

**Authors:** Lenis Chen-Edinboro, Daniel Johnson, Hannah Catalano, Tina Newsham

**Affiliations:** The University of North Carolina at Wilmington, Wilmington, North Carolina, United States; The University of North Carolina at Wilmington, Wilmington, North Carolina, United States; The University of North Carolina at Wilmington, Wilmington, North Carolina, United States; The University of North Carolina at Wilmington, Wilmington, North Carolina, United States

## Abstract

Music is an avenue for meaningful and life-long self-expression and well-being, especially for older adults. In this multiple case ethnographic study, we investigated connections between choral singing and wellness among people aged 67 to 80. We considered the effects of making music in six dimensions of holistic wellness: developmental, cognitive, physical, relational/social, emotional, and spiritual domains (Fullen, 2019). We interviewed six participants who engaged in shape note singing, a traditional American form of choral singing. We transcribed and analyzed data inductively and deductively, searching for themes within and across participants beginning with open coding to gain an overall understanding of participants’ perceptions and experiences. We then conducted cross-participant analysis using focused coding to determine three emergent themes that described physiological and psychological health benefits of shape note singing: musical engagement, emotional response, and interpersonal connections. We found that older people who participated in shape note singing experienced several physiological and psychological health benefits across the considered dimensions of holistic wellness. Implications are for gerontologists to use this or other forms of making music to promote engagement, self-expression, and interpersonal connections. Other insights are to include music-based social interactions to reduce loneliness and isolation and to promote holistic wellness. Fullen, M. C. (2019). Defining wellness in older adulthood: Toward a comprehensive framework. Journal of Counseling & Development, 97(1), 62-74.

